# Commentary on a recent article on the effects of the ‘Daily Mile’ on physical activity, fitness and body composition: addressing key limitations

**DOI:** 10.1186/s12916-019-1335-4

**Published:** 2019-05-22

**Authors:** Andy Daly-Smith, Jade L. Morris, Matthew Hobbs, Jim McKenna

**Affiliations:** 1 School of Sport, Leeds Beckett University, Headingley Campus, Headingley, Leeds, LS6 3QS UK; 2 The GeoHealth Laboratory, The Geospatial Research Institute, University of Canterbury, Christchurch, New Zealand

**Keywords:** The Daily Mile™, Physical activity, Primary schools, Children, Physically active learning, Classroom movement breaks, Lessons, Run-walk breaks

## Abstract

A recent pilot study by Chesham et al. in *BMC Medicine* established some initial effects of the Daily Mile™ using a quasi-experimental repeated measures design, with valid and reliable outcome assessments for moderate-to-vigorous physical activity, fitness and body composition. Their contribution is important and welcome, yet, alone, it is insufficient to justify the recent UK-wide adoption of the Daily Mile within the Childhood Obesity Plan. The study concluded that the Daily Mile had positive effects on moderate-to-vigorous physical activity, fitness and body composition, suggesting that intervention effectiveness was confirmed. However, only some of the significant limitations of the work were addressed. Herein, we identify and discuss six key limitations, which, combined, suggest a more tentative conclusion. In summary, evidence supporting the effectiveness of the Daily Mile is in its infancy and requires refinement to fully justify its widespread adoption. Further, we need to be cautious considering that the full range of its impacts, both positive and negative, remain to be fully established.

## Background

UK children are insufficiently active, with 60–80% failing to reach government guidelines on physical activity (PA) [1]. Consequently, PA promotion is high on the UK policy agenda [2], with schools being at the forefront of possible interventions due to their dominance of children’s waking hours. Schooltime, which is compulsory and highly sedentary [3], provides an excellent opportunity to generate bouts of both light and moderate-to-vigorous PA (MVPA) [4]. Three PA intervention modes during school time have grown in prominence, namely classroom movement breaks, physically active learning and run-walk breaks. The effects of classroom movement breaks and physically active learning programmes on PA, academic performance and cognition are well documented [5, 6]. Conversely, the effectiveness of run-walk programmes is yet to be confirmed.

Recently, Chesham et al. [7] reported a quasi-experimental repeated measures pilot study (including two schools) to assess the effects of the Daily Mile™ (TDM) – a school-based run-walk programme – on MVPA, fitness and body composition. This rigorous original study was based on a large initial sample size (*n* = 391), it employed a control group and focused on psychometrically validated outcome assessments. Nevertheless, various unacknowledged issues may have affected the outcome measures, data collection process and interpretation of the results.

This commentary outlines six key limitations – some of which are additional to those identified by Chesham et al. [7] or raised within the review process – to suggest a more cautious interpretation of the results. Collectively, and at this time, these issues raise concerns about the appropriateness of the study conclusion, namely that “*the Daily Mile intervention is effective at increasing levels of MVPA, reducing sedentary-time, increasing physical fitness and improving body composition. These findings have relevance for teachers, policymakers, public health practitioners, and health researchers*” [7].

### Six key limitations

#### Varied exposure durations create unequal dose–response conditions

Intervention participants were exposed to TDM for approximately 28 weeks (October to May), which is 2.5 times longer than the approximately 12 weeks (March to June) between the pre- to post-measurement period for the control group. While the authors controlled for age to account for varied start times, to our knowledge, this approach does not fully ameliorate such limitations.

#### Varied start and end times create unequal opportunity for benefits

The pre- and post-intervention measurements for the TDM group were taken in October (autumn) and May (summer), whereas the control measures were taken in March (spring) and June (summer). Seasonality [8] and weather conditions [9] impact PA levels. Generally, within the UK, MVPA levels are higher in spring and summer compared to autumn and winter [10, 11]. Controlling for the weather and season in the analyses could account for some of these differences [9]. Additionally, body fat levels also tend to be higher, and fitness lower, following the UK summer vacation [12]. Furthermore, baseline outcomes identified important between-group differences that may influence responsiveness – control participants had higher levels of MVPA (7/7 groups), fitness (6/7 groups), BMI z-scores (4/7 groups) and sum of skinfolds (6/7 groups). Although analyses controlled for age, these baseline characteristics, combined with varied exposure duration, may unduly influence study findings.

#### Aggregated intervention outcomes mask variable individual response

The standard approach of representing intervention effects through central tendency revealed that TDM increased MVPA by an average of 9.1 min; however, a visual count of the data in figure two A [7] revealed highly varied intervention effects. For example, in the intervention group, these ranged from an approximately 55 min/day decrease in MVPA to an increase of approximately 60 min/day. Furthermore, while 55% of the intervention participants had increased MVPA outcomes following the TDM intervention, 45% reduced their MVPA. Clearly, substantial numbers of children did not respond positively to TDM, with similar results as those found in the control group, where 44% improved their MVPA outcomes at follow-up and yet 56% of children had worsened outcomes. Therefore, in this context, it is not accurate to claim universal benefit. Future studies using larger sample sizes should use subgroup analyses to identify the characteristics of the participants who may and may not benefit from TDM [13].

#### Small samples limit the generalisation of results

The final intervention MVPA sample included only 17% of the original participants (*n* = 56) and 37% of original controls (*n* = 62). It is unwise to assume these children are representative of the full sample and this further limits the generalisation of outcomes. Although not essential, sensitivity analyses would have enabled comparisons of key subsample characteristics and established any bias arising from potential systematic attrition of individuals [14]. Where feasible, we recommend that future studies assess the effectiveness of TDM by recruiting larger samples across multiple schools. To account for the nested structure of pupils within classes within schools, multilevel modelling analyses is justified [15].

#### Variable approaches to using accelerometers reduces validity

Four key issues arose in relation to accelerometer use. First, data were collected in 60-s epochs, rather than the established 15-s epochs in the original cut-point validation study [16]. Current evidence suggests deviation from the validated cut-point epoch reduces the validity of PA outcomes [17]. Second, shorter epochs of ≤ 15 s more accurately assess the sporadic higher intensity activity present within children’s PA patterns [17, 18]. Third, while the use of five different accelerometer models was justified in the paper, it was not reported how consistently participants wore the same accelerometer model or unit in the pre- and post-assessments. Fourth, consistent with previous studies, 3 days of wear time were required to estimate accelerometer outcomes. However, these included weekend days when TDM was not delivered, meaning that MVPA outcomes influenced by weekend factors are not attributable to TDM. Related to this final point, it may have been useful to understand what happens to PA levels on days with and without TDM; previous studies have observed between-day compensatory effects for PA and sedentary behaviour [19].

#### Not confirming the treatment dose reduces confidence in the outcomes

The contribution of TDM was not directly confirmed using segmented day analysis to identify acute MVPA responses and outcomes. Further, no assessment of treatment fidelity occurred to assess how often TDM was implemented throughout the intervention period. Therefore, it is difficult to qualify the outcomes that relate directly to TDM.

### Summary and ways forward

TDM intervention is rapidly diffusing across the UK and internationally, driven by television commercials, celebrity endorsement and private funding. We commend Chesham et al. [7] for conducting the first pragmatic trial on the effectiveness of TDM. However, the cumulative impact of the limitations addressed in the original paper, supplemented by the issues we raise herein, support a more tentative endorsement of TDM than is offered in the original paper. Consequently, UK policymakers [2] and teachers who wish to adopt TDM should accept that they proceed while knowing that the full range of its impacts – both positive and negative – remain to be fully established.

To fill this evidence gap, high-quality translational studies that meet the standards for identifying effective prevention programmes are required [20]. Two such studies investigating different UK school-based run-walk programmes are currently in progress [21, 22]. In addition to establishing physiological impact, both studies seek to explore the efficacy of run-walk interventions to children’s well-being and long-term PA behaviours. To date, there has been limited dialogue around the potential polarising impact that run-walk interventions may have on children’s relationship with PA. For children who find steady-state aerobic exercise enjoyable, this may be positive; for others who dislike continuous non-cognitively challenging exercise, the experience may incubate the first stages of lifelong inactivity.

## Abbreviations

BMI: body mass index
MVPA: moderate-to-vigorous physical activity
PA: physical activity
TDM: The Daily Mile™
